# *In vitro* and *in vivo* evaluation of kenaf (*Hibiscus cannabinus* L.) as a roughage source for beef cattle

**DOI:** 10.5713/ajas.17.0871

**Published:** 2018-04-12

**Authors:** Seongjin Oh, David Tinotenda Mbiriri, Chaehwa Ryu, Kangheon Lee, Sangbuem Cho, Nag-Jin Choi

**Affiliations:** 1 Department of Animal Science, Chonbuk National University, Jeonju 54896, Korea; 2 Department of Animal Science, University of Zimbabwe, Harare 00263, Zimbabwe

**Keywords:** Carcass Characteristics, Growth Performance, Hanwoo, Kenaf, Roughage

## Abstract

**Objective:**

The goal of this study was to evaluate kenaf as a roughage source *in vitro* and its effects on meat quality of Hanwoo (Korean native) cattle.

**Methods:**

Three roughage materials, rice straw silage, ryegrass silage, and kenaf silage, were tested in a batch culture and feeding trial. Rumen fermentation parameters, including gas, pH, volatile fatty acid (VFA), and ammonia were analyzed. In the feeding trial, Hanwoo steers (373.5±5.1 kg, n = 36, 11 month of age) were divided into three feeding groups (n = 12 each). Animals were fed with each silage and concentrate until the fattening stage.

**Results:**

Crude protein, ether extract, and non-structural carbohydrates were greater in kenaf silage. Total gas production was higher in ryegrass silage, followed by kenaf silage and rice straw silage (p<0.05). Total VFA and individual VFA (acetate, propionate, and n-butyrate) were greater in kenaf silage than rice straw silage (p<0.05). *In vitro* dry matter digestibility showed a similar trend to that of total gas and VFA production; it was higher in ryegrass silage and lower in rice straw (p<0.05). Throughout the feeding trial, the rice straw silage group showed significantly greater average daily gain than did the others (p<0.05). The feed conversion ratio in the group fed kenaf silage was significantly greater than that of others (p<0.05). No significant differences were observed in yield or quality traits, including carcass weight, ribeye area, backfat thickness, and scores for marbling, meat color, and fat color (p>0.05).

**Conclusion:**

The results indicated that no negative effects on growth performance and carcass characteristics occurred across treatments. Therefore, kenaf could be substituted for rice straw, which is most widely used as a roughage source in Korea.

## INTRODUCTION

Increasing the livestock feed resource base is vital in a world where livestock production is increasing and intensifying. A continuous quest to identify alternative feed resources relieves pressure on conventional feed resources: primarily grain and oil seeds. Kenaf is one of the fodder crops that has a place in the feed resource-base inventory.

Kenaf (*Hibiscus cannabinus* L.) is an annual herbaceous plant native to southern Asia and is traditionally grown for its fiber. It has wide applications in the manufacturing industry [1] and that consequently prompted its adoption under various geographical and climatic conditions throughout the world. The nutrient content available for animals, particularly crude protein content, is known to vary depending on the region of inhabitation, climate, harvest time, and the portion of the plant used. It was reported that crude protein content in leaves could reach up to 20% crude protein during its immature stage [2]. As the plant matures, however, crude protein content in the stems and leaves declines. Kenaf seeds have also been used as a protein supplement for ruminant livestock on pasture [3]. The crude protein content of kenaf seeds ranges from 24% to 35% [4].

Roughage, in addition to supplying nutrients, is important in maintaining normal rumen function crucial to the health of the animal and also better meat quality and dairy products. A number of studies have been conducted to evaluate kenaf as a forage source for ruminant animals [5–7]. However, there has been little study concerning the effects of kenaf feeding on Hanwoo steer, Korean native steer. We therefore designed this study to evaluate kenaf *in vitro* and its effects on the meat quality of Hanwoo steer.

## MATERIALS AND METHODS

### Rumen fluid and donor cattle management

Rumen contents were obtained from ruminally cannulated cross-bred cattle (n = 2, 700±50 kg) fed once daily with 10 kg rice straw and 3 kg concentrate at a farm located in Buyeo District, Korea. Mineral block and water were made freely available. Rumen contents from the two cattle were mixed and then squeezed through four layers of cheese cloth to remove feed particles and to obtain rumen fluid. The strained rumen fluid was placed in a bottle flushed with N_2_ gas, then immediately transferred to the laboratory.

### *In vitro* rumen fermentation

Three roughage sources, rice straw silage, ryegrass silage, and kenaf silage, were tested to compare the effectiveness of kenaf silage as a new forage material. *In vitro* fermentation was performed as described by Tilley and Terry [8]. Briefly, 50 mL rumen inoculant was mixed with 0.5 g substrate and then anaerobically incubated. Incubation time was 24 h. Rumen fluid was mixed with artificial saliva [9] in a 1:4 ratio. Each fermentation bottle was immediately sealed with a butyl rubber cap and an aluminum cap. Rumen inoculant was incubated with 0.5 g substrate at 39°C. After incubation, the volume of fermentation gas in the headspace was measured using a glass syringe, and it was then collected in aluminum packs for gas analysis. Rumen fluid was then sampled and kept in a freezer at −30°C for analysis of rumen fermentation parameters including pH, volatile fatty acid (VFA), and ammonia.

### Chemical analysis

We analyzed chemical components of experimental diet using the methods of the AOAC [10] and Van Soest et al [11]. Sample analysis was carried out as previously described by Mamvura et al [12]. Briefly, total gas production was measured using a needle-attached glass syringe. Ruminal pH was measured using a pH meter (S20 Seven Eazy, Mettler-Toledo, Schwerzenbach, Zurich, Swltzerland). VFAs were analyzed as previously described by Erwin et al [13]. Rumen fluid supernatant was mixed with 25% metaphosphoric acid in a 5:1 ratio. The sample mixture was incubated for 30 min at room temperature, then centrifuged at 13,000 rpm. VFA were detected using a gas chromatography analyzer (HP7890, Agilant, CA, USA) equipped with a capillary column (Nukol, 0.25 mm i. d.×0.25 μm film×30 m length, SUPELCO, Bellefonate, PA, USA). Temperature conditions were 220°C, 180°C, and 200°C for injector, oven, and detector, respectively. Ammonia was analyzed using the indophenol reaction noted by Cheney and Marbach [14]. *In vitro* dry matter digestibility (IVDMD) was calculated as described by Moore [15].

### Feeding trial using kenaf silage

We used kenaf silage in a feeding trial using Hanwoo steers (373.5±5.1 kg, n = 36, 11 month of age). Rice straw silage and ryegrass silage were also tested with kenaf silage as reference silage. Experimental animals were divided into three groups (n = 12 each) with the same initial body weight. We defined three growth stages: growing period (7 to 12 months of age), early fattening period (13 to 18 months of age), and late fattening period (19 to final month of age). Animals were fed with each silage (Table 1) and concentrate containing crude protein (17%, 14%, and 12.5% for each growth stage, respectively), ether extract (3.0%, 3.5%, and 3.5%), crude fiber (13%, 15%, and 15%), crude ash (12%, 12%, and 12%), and total digestible nutrients (72.0%, 74.5%, and 76.5%). Mineral block and water were freely available. Daily feed intake was checked. The feeding trial was performed at an experimental farm located in Jeong-eup city, Korea.

### Statistical analysis

Rumen fermentation parameters from the batch culture, including gas production, pH, VFA, and ammonia, as well as IVDMD, growth performance, carcass characteristics, and meat quality in the feeding trial were averaged and subjected to an analysis of variance using the general linear model procedure of SPSS (version 12). Duncan’s multiple range test was employed for multiple comparisons. Statistical significance was considered at p<0.05.

## RESULTS

Nutrient contents of silage used in this study are summarized in Table 1. Kenaf silage had greater moisture content compared to that of the others. Crude protein, ether extract, and non-structural carbohydrates (NSCs) were greater in kenaf silage, whereas neutral detergent fiber (NDF) and acid detergent fiber (ADF) were lower than that of other silages. Kenaf silage contained higher levels of NSC (22.2%) than did rice straw silage (11.0%) or ryegrass silage (16.0%). Kenaf silage was greater in NSC and crude protein than rice straw silage and ryegrass silage (Table 1). Total gas production was greater in ryegrass silage, followed by kenaf silage and rice straw silage (p<0.05). Moderate levels of ruminal pH were observed in all three silages. Total VFA and individual VFA (acetate, propionate, and n-butyrate) were greater in kenaf silage than in rice straw silage (p<0.05). Ryegrass silage showed the highest VFA profile among silages tested (p<0.05). A relatively high level of ammonia nitrogen was observed in kenaf silage, followed by rice straw silage and ryegrass silage (p<0.05). IVDMD showed a similar trend to that of total gas and VFA production, being higher in ryegrass silage and lower in rice straw (p<0.05).

*In vitro* rumen fermentation parameters indicated that kenaf silage supported normal rumen function and was actually superior to rice straw silage in almost all parameters (Table 2). Although kenaf silage was second to ryegrass silage, it resulted in higher (p<0.05) ammonia nitrogen production.

Growth performance of Hanwoo steers fed the three different silages is as shown in Table 3. Growth performance in the growing period was partially omitted because animals refused kenaf silage. In the early fattening period, no difference was observed in average daily gain (ADG) and final body weight (p>0.05). The kenaf silage group had a greater feed conversion ratio than did the other silage groups (p<0.05). In the late fattening period, differences in feed efficiency was more apparent. ADG was lower in the kenaf silage group than the rice straw silage group (p<0.05), and the feed conversion ratio was significantly greater in kenaf silage (16.92) than in the rice straw silage (12.06) and ryegrass silage (14.48) (p< 0.05). There were no differences in final body weight (p>0.05). Throughout the feeding trial, the rice straw silage group showed significantly greater ADG than did the others (p<0.05). The feed conversion ratio in the group fed kenaf silage was significantly greater than that of the others (p<0.05). Total intake of roughage in the group fed kenaf silage was significantly greater than that of the others (p<0.05).

Although kenaf silage intake by Hanwoo steers was lower (p<0.05) than the other test silages throughout the feeding trial, the final weights for the various stages, growth, early fattening, and late fattening phases, did not differ (p>0.05) (Table 3). Average daily weight gain for the kenaf group was lower (p< 0.05) than that of the other silage groups during the growth and early fattening stages. During the early fattening stage, steers supplemented with kenaf silage had higher (p<0.05) concentrate feed intake than the others. It was only in the late fattening phase that the kenaf group had the highest feed conversion ratio.

Effects of kenaf feeding to Hanwoo steers on carcass characteristics is shown in Table 4. No significant differences were observed in yield and quality traits, including carcass weight, ribeye area, backfat thickness, and scores of marbling, meat color, and fat color (p>0.05). Most of the animals in the kenaf silage feeding group were C rank, the lowest yield grade level. Different silage feeding did not affect to meat quality (data not shown). Supplementing Hanwoo steers from the growing to the late fattening period (a period spanning just over 4 months) did not have any effect (p>0.05) on meat quality (Table 4).

## DISCUSSION

The present study was conducted to provide practical information on the use of kenaf as a feed source. Kenaf used in this study was finely cut (<15 mm) and ensiled before experimental use because fresh kenaf was not suitable for direct feeding to animals because i) woody stems are highly lignified, which is closely related to poor rumen degradability [16,17], and ii) high moisture content has negative effects on long-term storage [18]. Rice straw silage and ryegrass silage are widely used as roughage sources in Korea, and rice straw is generally regarded as low in nutrient content [19].

Kenaf silage had 6.6% crude protein, greater than that of the other silages. This value, however, is relatively lower than that previously reported, in which crude protein of various kenaf cultivars varied in the range of 6.9% to 13.4% [20]. NDF and ADF contents in kenaf are known to be lower than that of other roughage, including rice straw and ryegrass [21]. Although kenaf showed potent nutritive value, particularly in crude protein, it is suggested that factors such as growth stage, and part and cultivar affecting nutritional quality should be carefully considered when planning to use kenaf in any form of roughage [21–23].

Overall, the *in vitro* rumen fermentation parameters in kenaf silage were more potent than that of rice straw silage. Greater gas and VFA production (total, acetate, and propionate) and IVDMD in kenaf silage than rice straw silage can be attributed to the greater proportion of NSC in kenaf silage. Although excessive NSC levels in the diet can lower ruminal pH and enzyme production to degrade fiber, adequate levels of NSC stimulate rumen microbial activity through which fiber digestion could be increased [24]. Different NSC levels in the diet scarcely affect ammonia levels [25], indicating greater ammonia in kenaf silage can be attributed to crude protein levels [26]. As seen for rumen fermentation parameters, the nutrient content of kenaf silage did not negatively affect rumen fermentation and feed utilization.

In the feeding trial, final body weight was not affected by the three experimental silages. All groups consumed similar levels of concentrate throughout the growth stage, whereas roughage intake was lower for the kenaf silage (Table 3), indicating a decrease of energy density because of the high moisture content of kenaf silage. Therefore, actual NSC intake of the rice straw silage group and kenaf silage group would be similar.

There was no statistical difference observed in carcass characteristics with different silages (p>0.05) for yield traits, including carcass weight, ribeye area, backfat thickness, and yield index. According to a meta-analysis on the effects of roughage quality on Hanwoo meat quality, the following traits can be affected: carcass weight, ribeye area, and yield index [27].

The results of the present study indicated that no negative effects on growth performance and carcass characteristics were founded across the treatments. Therefore, kenaf could be substituted for rice straw, which is most widely used as a roughage source in Korea.

## Figures and Tables

**Table 1:** Chemical composition of experimental diet (% dry matter basis)

Content	Rice straw silage	Ryegrass silage	Kenaf silage
Dry matter	84.6	84.3	23.5
Crude protein	5.2	5.4	6.6
Ether extract	2.4	2.1	3.8
Neutral detergent fiber	71.4	69.0	60.0
Acid detergent fiber	46.6	44.9	43.1
Crude ash	10.0	7.5	7.4
Non-structural carbohydrate	11.0	16.0	22.2

**Table 2:** Effect of kenaf silage supplementation on *in vitro* rumen fermentation

Parameters	Rice straw silage	Ryegrass silage	Kenaf silage	SEM	p-value
Total gas (mL)	30.67^c^	53.30^a^	37.67^b^	3.29	<0.001
pH	6.66^a^	6.56^c^	6.61^b^	0.01	<0.001
Total VFA (mM)	47.58^c^	60.72^a^	52.73^b^	1.82	<0.001
Acetate (mM)	25.50^c^	32.75^a^	29.98^b^	1.01	<0.001
Propionate (mM)	9.41^c^	13.20^a^	9.97^b^	0.56	<0.001
n-Butyrate (mM)	4.90^b^	5.59^a^	4.87^b^	0.11	<0.001
Ammonia-N (mg/dL)	3.40^b^	2.87^c^	7.04^a^	0.62	<0.001
IVDMD (%)	24.23^c^	35.06^a^	29.67^b^	1.54	0.001

SEM, standard error of mean; VFA, volatile fatty acid; IVDMD, *in vitro* dry matter digestibility.

abc Different superscript in same row means significantly different (p<0.05).

**Table 3:** Effect of kenaf silage feeding on growth performance of Hanwoo steers

Contents	Rice straw silage	Ryegrass silage	Kenaf silage	SEM	p-value
Initial body weight (kg)	373.06	378.42	367.42	4.78	0.683
Growing period (11–12 months)					
Average daily gain (kg/d)	1.05^a^	0.91^ab^	0.77^b^	0.04	<0.05
Feed conversion ratio	9.40^ab^	10.34^a^	8.33^b^	0.33	<0.05
Feed intake (kg DM/d)					
Concentrate	4.78	4.78	4.78	0.01	1.000
Roughage (silage)	4.65^a^	4.63^a^	1.42^b^	0.10	<0.05
Final body weight (kg)	411.33	416.42	405.44	4.96	0.665
Early fattening period (12–19 months)					
Average daily gain (kg/d)	1.00	0.98	0.98	0.02	0.931
Feed conversion ratio	9.10^a^	9.26^a^	8.02^b^	0.21	<0.05
Feed intake (kg DM/d)					
Concentrate	6.70^b^	6.72^b^	6.85^a^	0.02	<0.05
Roughage (silage)	2.18^a^	2.21^a^	0.92^b^	0.03	<0.05
Final body weight (kg)	541.25	543.92	533.13	5.49	0.699
Late fattening period (19–27 months)					
Average daily gain (kg/d)	0.69^a^	0.56^ab^	0.52^b^	0.03	<0.05
Feed conversion ratio	12.06^b^	14.48^b^	16.92^a^	0.99	0.130
Feed intake (kg DM/d)					
Concentrate	6.74	6.61	6.69	0.03	0.152
Roughage (silage)	1.27^a^	1.24^a^	0.57^b^	0.01	<0.05
Final body weight (kg)	745.00	718.25	709.44	8.58	0.220
Whole period (11–27 months)					
Average daily gain (kg/d)	0.86^a^	0.78^b^	0.77^b^	0.02	<0.05
Feed conversion ratio	9.94^b^	10.89^b^	11.22^a^	0.20	<0.05
Feed intake (kg DM/d)					
Concentrate	6.57^ab^	6.51^a^	6.60^b^	0.02	0.094
Roughage (silage)	1.92^a^	1.92^a^	0.78^b^	0.02	<0.05

SEM, standard error of mean; DM, dry matter.

a,b Different superscript in same row means significantly different (p<0.05).

**Table 4:** Effect of kenaf silage feeding to Hanwoo steers on carcass yield and meat quality1)

Indices	Rice straw silage	Ryegrass silage	Kenaf silage	SEM	p-value
Carcass yield					
Carcass weight (kg)	460.9	462.3	458.0	5.75	0.953
Ribeye area (cm^2^)	99.8	100.3	90.6	2.02	0.072
Backfat thickness (mm)	16.3	13.8	16.5	0.81	0.328
Yield index	63.2	64.8	61.9	0.60	0.143
Yield grade (A:B:C, %)	8:50:42	25:33:42	0:17:83	-	-
Meat quality					
Marbling score	6.75	6.92	5.63	0.28	0.100
Meat color score	4.67	4.67	4.81	0.07	0.619
Fat color score	3.00	3.33	3.00	0.09	0.212
Quality grade (1++:1+:1:2)	33:50:17:0	42:42:16:0	33:25:25:17	-	-

SEM, standard error of mean.

1) Carcass traits were determined according to Korean carcass grading standard by an official grader.
